# Association Between a Temporary Reduction in Access to Health Care and Long-term Changes in Hypertension Control Among Veterans After a Natural Disaster

**DOI:** 10.1001/jamanetworkopen.2019.15111

**Published:** 2019-11-13

**Authors:** Aaron Baum, Michael L. Barnett, Juan Wisnivesky, Mark D. Schwartz

**Affiliations:** 1 Department of Health System Design and Global Health, Arnhold Institute for Global Health, Icahn School of Medicine at Mount Sinai, New York, New York; 2 Veterans Affairs New York Harbor Healthcare System, New York, New York; 3 Department of Health Policy and Management. Harvard T. H. Chan School of Public Health, Boston, Massachusetts; 4 Department of Medicine, Icahn School of Medicine at Mount Sinai, New York, New York; 5 Department of Population Health, New York University School of Medicine, New York, New York

## Abstract

**Importance:**

Temporary disruptions in health care access are common, but their associations with chronic disease control remain unknown.

**Objective:**

To evaluate whether long-term changes in chronic disease control were associated with a temporary 6-month decrease in access to health care services.

**Design, Setting, and Participants:**

This cohort study examined the long-term changes in chronic disease control associated with the 6-month closure of the Manhattan facility of the Veterans Affairs (VA) New York Harbor Healthcare System after superstorm Sandy, which caused a significant disruption in health care access for veterans in the region. Electronic health records from the VA Healthcare System between October 29, 2010, and October 29, 2014, were used to identify a total of 81 544 veterans who were and were not exposed to the 6-month closure of the VA Manhattan Medical Center after superstorm Sandy. Of those, 19 207 veterans were included in the exposed cohort and 62 337 were included in the nonexposed control cohort, which included veterans who were equally exposed to the storm but who retained regular access to health care from 3 VA medical centers (Brooklyn and the Bronx in New York and New Haven in Connecticut) during and after the storm. A difference-in-differences analysis was used to assess within-patient changes in chronic disease control over time between a cohort that was exposed to decreased health care access compared with a similar cohort that was not exposed to decreased access. All analyses adjusted for individual demographic and socioeconomic characteristics, between–zip code differences, and common time trends. Data analyses were conducted between February 1, 2016, and September 30, 2019.

**Exposure:**

The 6-month closure of the VA Manhattan Medical Center after superstorm Sandy on October 29, 2012.

**Main Outcomes and Measures:**

The outcomes measured were uncontrolled blood pressure (defined as mean blood pressure per patient per quarter >140/90 mm Hg), uncontrolled diabetes (defined as mean hemoglobin A_1c_ per patient per quarter >8%), uncontrolled cholesterol (defined as mean low density lipoprotein per patient per quarter >140 mg/dL), and patient weight.

**Results:**

Among the 81 544 veterans included in the study, the mean (SD) age was 62.1 (17.6) years, and 93.6% were men, 62.7% were white, and 31.8% were black. At the 3-month midpoint of the 6-month facility closure of the VA Manhattan Medical Center, an absolute decrease of 24.8% (95% CI, −26.5% to −23.0%; *P* < .001) was observed in the percentage of veterans who had any VA primary care visit per quarter compared with a baseline of 47.8% before the closure (relative decrease, 51.9%; 95% CI, −55.4% to −48.1%; *P* < .001). One year after the facility reopened, no differential change was observed in the percentage of patients with a primary care visit between the exposed vs nonexposed cohorts (absolute decrease, −0.1%; 95% CI, −1.5% to 1.4%; *P* = .94); however, patients in the exposed cohort were 25.9% more likely to have uncontrolled blood pressure than patients in the nonexposed cohort (unadjusted increase, 5.5% in the exposed cohort vs 1.3% in the nonexposed cohort; adjusted absolute increase, 5.0%; 95% CI, 3.5%-6.0%; *P* < .001). Two years after superstorm Sandy, patients in the exposed cohort were 10.9% more likely to experience uncontrolled blood pressure than those in the nonexposed cohort (unadjusted increase, 5.2% in the exposed cohort vs 3.5% in the nonexposed cohort; adjusted absolute increase, 2.1%; 95% CI, 0.5%-3.6%; *P* < .001). Compared with the nonexposed cohort, the exposed cohort also experienced a decrease in filled medication prescriptions per patient per quarter of 6.9% during the facility closure (absolute decrease, −0.7 prescriptions filled per patient per quarter; 95% CI, −0.9 to −0.5; *P* < .001) and of 2.2% a year after the facility reopened (absolute decrease, −0.2 prescriptions filled per patient per quarter; 95% CI, −0.4 to −0.1; *P* = .04). No differential changes were observed in uncontrolled diabetes, uncontrolled cholesterol, or patient weight.

**Conclusions and Relevance:**

In this study, a temporary period of decreased access to health care services was associated with increased rates of uncontrolled hypertension, but not with increased rates of uncontrolled diabetes or hyperlipidemia, more than 1 year after the Manhattan VA facility reopened. Temporary gaps in access to health care may be associated with long-term increases in uncontrolled blood pressure among patients with hypertension.

## Key Points

**Question:**

What long-term changes in chronic disease control were associated with a temporary period of decreased access to health care services among veterans after a natural disaster?

**Findings:**

In this cohort study of 81 544 veterans receiving health care services in the Veterans Affairs Healthcare System, veterans exposed to the 6-month closure of the VA Manhattan Medical Center after superstorm Sandy experienced increases in uncontrolled hypertension that persisted for 24 months. The decrease in access to care was not associated with increased rates of uncontrolled diabetes or hyperlipidemia.

**Meaning:**

Temporary decreases in health care access may be associated with lasting increases in uncontrolled blood pressure among patients with hypertension.

## Teaser

This cohort study examines whether long-term changes in chronic disease control were associated with a 6-month decrease in access to health care services among veterans in New York City after superstorm Sandy in October 2012.

## Introduction

Many US residents experience temporary disruptions in access to health care services every year. For example, lapsed private health insurance coverage often follows job loss,^1,2^ and 30% of Medicaid beneficiaries experience gaps in insurance coverage owing to eligibility changes, with a mean duration of 6 months.^3,4^ Although positive effects of access to health care through acquisition of health insurance have been reported extensively,^5^ the effect of decreased health care access on patient health remains unknown.

Patients with a chronic illness may be at a high risk of negative health consequences from temporary gaps in health care access.^6,7^ Hypertension, which has been diagnosed in over one-third of the adult US population, including more than 65% of adults aged 60 years and older, is a challenging chronic illness to manage during periods of disrupted access to care.^8,9^ For example, individuals with hypertension reported lower use of health care services and poorer health status immediately after job loss.^10^

Given the frequency with which individuals and communities experience disruptions in health care access, it is crucial to understand the consequences of intermittent gaps in access among patients with chronic illness. Unfortunately, these consequences are difficult to isolate because episodes of decreased health care access are often associated with many other factors that can affect health. Observational studies suggest that greater access or proximity to primary care is associated with improved health status,^11,12,13^ although those studies could not rule out confounding from unobserved factors that may vary with access to both health care and health outcomes. Data from permanent hospital closures, which have been used as proxies to measure the consequences of decreased access to inpatient and emergency department facilities, have indicated an association between hospital closure and increased short-term mortality among patients with time-sensitive acute conditions^14,15^; however, hospital closures are often associated with local factors, such as economic conditions or poor performance, that can play a role in both facility closures and health outcomes.

To address this evidence gap, we examined the temporary closure of the Manhattan facility of the Veterans Affairs (VA) New York Harbor Healthcare System following superstorm Sandy, the largest Atlantic hurricane on record. Damages from the October 29, 2012, storm forced the Manhattan VA Medical Center, which includes a large outpatient facility, to close all operations for 6 months, requiring patients to visit other VA centers for health care services. This closure resulted in a temporary period of overall decreases in outpatient VA health services use among veterans who usually received care from the Manhattan facility.^16,17,18^ We used this closure as a natural experiment to examine the association between a temporary decrease in health care access and long-term control of hypertension, diabetes, and hypercholesterolemia.

## Methods

This study followed the Strengthening the Reporting of Observational Studies in Epidemiology (STROBE) reporting guidelines for cohort studies.^19^ We obtained data on 273 024 veterans who visited the VA facilities in Manhattan, Brooklyn, and the Bronx, New York, and New Haven, Connecticut, 1 or more times between October 29, 2010, and October 29, 2014. We considered a veteran’s usual place of care to be a given VA facility if the veteran visited the facility for 25% or more of his or her health care appointments before superstorm Sandy. In sensitivity analyses, we examined 2 alternate thresholds for assigning a patients' usual place of care, which were respectively based on where patients had the plurality or majority of their health care visits before the storm. These analyses indicated that a total of 81 544 adult veterans used the VA Manhattan, Brooklyn, Bronx, or West Haven medical center as their usual place of care before the storm (between October 29, 2010, and October 29, 2012). We used data from nationally integrated electronic health records stored in the Corporate Data Warehouse of the US Department of Veterans Affairs Health Administration Office of Information and Technology. The study was approved by the institutional review board of the VA New York Harbor Healthcare System with a waiver of informed consent because the research involved minimal to no risk for participants and could not be practicably conducted without a waiver.

### Selection of Exposed and Nonexposed Cohorts

The exposed cohort comprised 19 207 veterans who used the Manhattan VA Medical Center as their usual place of care before the storm on October 29, 2012, and thus experienced decreased access to health care services during the facility’s 6-month closure. The primary exposure was defined as an indicator variable equal to 1 if an individual was assigned to the Manhattan facility before the storm and equal to 0 if an individual was not. The nonexposed control cohort comprised 62 337 individuals who were assigned to the VA Bronx, Brooklyn, or West Haven medical centers and who did not experience decreased health care access.

Because the storm surge destroyed thousands of homes and left millions of people across multiple states without electricity,^20^ leading to quasi-random variation in exposure to household flooding, we performed secondary analyses to examine the association between exposure to reduced health care access and outcomes among the subpopulation who experienced flooding from the storm surge in addition to decreased access (eMethods in the Supplement).

The primary dependent variable was uncontrolled blood pressure (BP), defined as a binary variable equal to 1 when a patient's mean BP exceeded 140/90 mm Hg in a given quarter-year and equal to 0 otherwise. Secondary dependent variables included mean quarterly systolic and diastolic BP, uncontrolled type 2 diabetes (defined as mean quarterly hemoglobin A_1c _>8% [to convert to proportion of hemoglobin, multiply by 0.01]), uncontrolled cholesterol (defined as mean quarterly low-density lipoprotein level >140 mg/dL [to convert to millimoles per liter, multiply by 0.0259]), and patient weight. We also examined health care service use, including any primary care visit (identified using standardized VA stop codes)^21,22,23^ or inpatient admission per quarter, and number of prescription medications filled per quarter.

As covariates, we included sex, age, self-reported race (white, black, Asian, American Indian or native Alaskan, native Hawaiian or other Pacific Islander, unknown, and declined to answer), self-reported ethnicity (Hispanic, non-Hispanic, unknown, and declined to answer), marital status (married, divorced, separated, widowed, and never married), combat history, eligibility status for VA benefits (service connected ≥50%, service connected <50%, not service connected, and other), zip code, and any diagnosis of hypertension or diabetes before the storm (defined as ≥1 diagnosis recorded in the VA system before the storm). Race and ethnicity were reported by participants during the regular course of care (with options defined by the participant) and were used to compare demographic characteristics of the exposed and nonexposed cohorts before the storm and over time.

### Statistical Analysis

We used a difference-in-differences analysis^24^ with linear regression models to assess quarterly within-subject changes in outcomes in the 2 years before and after the storm among exposed patients assigned to the Manhattan VA facility compared with changes over the same period among nonexposed patients assigned to the 3 other VA facilities. To minimize potential bias owing to greater missing data during the facility closure among exposed vs nonexposed patients, we excluded the facility closure period and defined the period after the Manhattan VA facility reopened as the poststorm period. The key independent variables in the model were interactions between the Manhattan VA assignment (exposure) and each quarter-year period. The estimated coefficients on these interaction terms describe the average differential change in each outcome for exposed vs nonexposed patients over time. We included individual fixed effects in our model to control for both observable and unobservable time-invariant individual factors, such as baseline health status, that may have been associated with exposure or missing data. We also adjusted for quarter-year fixed effects and zip-code-of-residence fixed effects. These fixed effects controlled for observable and unobservable differences between local geography at the zip code level and time trends in the outcome that were common to both the exposed and nonexposed cohorts. Huber-White robust SEs were clustered at the zip code level.

To test a critical difference-in-differences assumption that the exposed and nonexposed cohorts shared a parallel prestorm trend in health outcomes, we tested for any association between exposure and outcomes in the prestorm period by including prestorm quarter-year periods interacted with a Manhattan VA assignment indicator.

We conducted subgroup analyses to determine whether the association between exposure and health outcomes differed among individuals who had vs did not have a prestorm diagnosis of hypertension or who were vs were not older than the sample’s median age (65 years, thus eligible for Medicare), and we assessed the effect of joint exposure to decreased access to care and flooding using the same design. Statistical tests were 2-sided with a significance threshold of *P* < .05; when *t* tests were used, they were unpaired. All data analyses were performed using Stata software, version 14 (Stata Corp), and were conducted between February 1, 2016, and September 30, 2019.

### Sensitivity Analyses

We conducted several sensitivity analyses to examine the robustness of our results. Two important concerns were differential attrition (ie, disproportionately more missing data among the exposed vs the nonexposed cohort) or compositional changes (ie, different types of patients leaving the sample in the exposed vs the nonexposed cohort) associated with exposure. First, we tested for differences in quarterly follow-up rates (the number of patients with at least 1 VA visit per quarter) and cohort composition (the baseline demographic characteristics and diagnosis histories of patients with any visit in each quarter) across the exposed vs nonexposed groups. Second, we quantified the maximum extent to which differential attrition could bias our results by assuming all excess attrition within the exposed cohort was caused by the departure of its healthiest patients.^25^ Third, we repeated the analysis for balanced panels of patients with 1 or more VA appointment, BP measurements, hemoglobin A_1c_ values, lipid panels, and recorded patient weights, respectively, both before and after the storm. Fourth, we ran the main analysis using alternative nonexposed control groups with characteristics more similar to the exposed group by using 1:1 coarsened exact matching^18^ to construct a matched control group with prestorm demographic characteristics and diagnosis histories similar to the exposed group, and by restricting the control group to patients assigned to a VA facility in New York City (excluding patients assigned to the West Haven, Connecticut, facility).

We performed additional analyses to assess the sensitivity of our results to alterations in variables and the statistical model. We examined a more stringent definition of uncontrolled BP, in which the binary primary dependent outcome variable was set equal to 1 only for patients who had at least 2 BP measurements greater than 140/90 mm Hg recorded within a 6-month period. In addition to individual and zip code fixed effects, we included fixed effects for zip code interacted with quarter-year to flexibly adjust for time-varying geographic factors, such as exposure to the storm surge, that may have differed across the exposed and nonexposed cohorts. Finally, we performed regression analyses with standard errors clustered at the VA facility level, using the wild-bootstrap *t* procedure, a conservative method used when there are few clusters across which exposure varies.^26^

## Results

### Population

Among 81 544 veterans included in the study, the mean (SD) age was 62.1 (17.6) years, and 93.6% of veterans were men, 62.7% were white, and 31.8% were black (Table 1). Overall, 70 813 patients (86.8%) had 1 or more BP measurements recorded. Before the storm, 67.9% of the study population had at least 1 recorded diagnosis of hypertension. Patients in the exposed cohort were less likely to be married (28.1% vs 43.7%; *P* < .001) and to have a diagnosis of hypertension (63.3% vs 69.4%; *P* < .001) or diabetes (25.7% vs 28.7%; *P* < .001) than patients in the nonexposed cohort. The probability that an individual was exposed to the storm surge was similar across cohorts that were differentially exposed to the facility closure (56.4% in the exposed cohort vs 53.4% in the nonexposed cohort; *P* = .26; eFigure 1 in the Supplement).

**Table 1.** Baseline Characteristics of Veterans Across VA Facilities

Characteristic	No. (%)		
	Total Population (N = 81 544)	Exposed Cohort (n = 19 207)	Nonexposed Cohort (n = 62 337)
Male	76 335 (93.6)	17 825 (92.8)	58 510 (93.9)
Age, mean (SD), y	62.1 (17.6)	60.5 (17.8)	62.5 (17.2)
Race			
White	47 046 (62.7)	10 016 (55.7)	37 030 (64.9)
Black	23 886 (31.8)	6815 (37.9)	17 053 (29.9)
Ethnicity			
Non-Hispanic	67 286 (85.5)	15 556 (83.0)	51 730 (86.2)
Married	32 001 (40.0)	5294 (28.1)	26 707 (43.7)
Combat history	12 834 (17.0)	2965 (16.4)	9869 (17.2)
VA eligibility^**a**^			
Service connected	19 006 (23.4)	4544 (23.8)	14 462 (23.3)
≤50% Service connected	15 496 (19.1)	3701 (19.3)	11 795 (19.0)
Not service connected	41 634 (51.3)	9330 (48.8)	32 304 (52.0)
Health status			
Hypertension	55 280 (67.9)	12 151 (63.3)	43 229 (69.4)
Diabetes	22 858 (28.0)	4937 (25.7)	17 921 (28.7)
Exposure			
Storm surge in zip code^**b**^	43 768 (54.1)	10 703 (56.4)	33 065 (53.4)

Abbreviation: VA, Veterans Affairs.

^a^ Service connected was determined by the VA based on the extent to which a veteran's injuries and illnesses were incurred or aggravated during active military service and varies with the degree of disability.

^b^ Storm surge in zip code was expressed as a binary variable equal to 1 if a patient’s zip code of residence had any spatial overlap with the Federal Emergency Management Agency Modeling Task Force’s determination of the extent of water inundation from superstorm Sandy and equal to 0 otherwise (eMethods in the Supplement).

**Figure 1.**
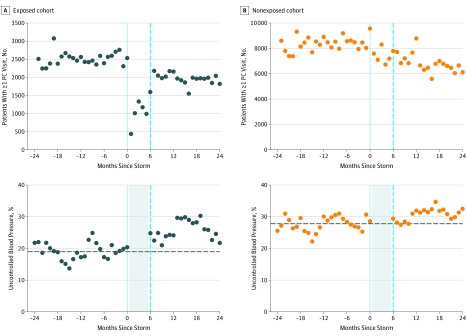
Unadjusted Rates of Primary Care Visits and Uncontrolled Blood Pressure Among Exposed vs Nonexposed Cohorts The exposed cohort comprised veterans who used the Manhattan Veterans Affairs (VA) Medical Center as their usual place of care before superstorm Sandy and who experienced decreased access to health care services during the facility's closure. The nonexposed control cohort comprised veterans who were assigned to the VA Bronx, Brooklyn, or West Haven medical centers and who did not experience decreased health care access after superstorm Sandy. PC indicates primary care.

The percentage of patients with 1 or more primary care visit or inpatient admission per quarter was similar in the exposed and nonexposed cohorts before the closure of the Manhattan VA facility and after the facility reopened 6 months later (Figure 1; eTable 1 in the Supplement). We found no evidence to indicate systematic differences in the types of patients remaining in the sample across the exposed and nonexposed cohorts (eFigure 2 in the Supplement).

### Use of VA Facilities

The unexpected closure of the Manhattan VA Medical Center caused a temporary, nearly complete loss of health care service at the facility for 6 months (eFigure 3 in the Supplement). Individuals assigned to the Manhattan VA facility experienced an immediate and large decrease in the probability of a visit to any VA health care professional during the 6-month closure. At the 3-month midpoint of the 6-month facility closure, an absolute decrease of 24.8% (95% CI, −26.5% to −23.0%; *P* < .001) was observed in the percentage of patients with any primary care visit among the exposed group compared with the nonexposed group; based on a prestorm baseline of 47.8%, this reduction represents a relative decrease of 51.9% (95% CI, −55.4% to −48.1%; *P* < .001). The percentage of patients with any inpatient admission to a VA facility in that quarter decreased by 2.4% (95% CI, −3.0% to −1.8%; *P* < .001) from a baseline of 6.6%, a relative decrease of 36.7% (95% CI, −45.5% to −27.3%, *P* < .001). The number of medication prescriptions filled per patient in that quarter decreased by 6.9% (absolute decrease, -0.7 prescriptions per patient per quarter; 95% CI, −0.9 to −0.5; *P* <.001), from a prestorm baseline of 9.1 medication prescriptions filled (eTable 1 in the Supplement).

One year after the facility reopened, no differential change was observed between the exposed vs nonexposed groups in the percentage of patients with a primary care visit (absolute decrease, −0.1%; 95% CI, −1.5% to 1.4%; *P* = .94) or inpatient admission (absolute increase, 0.7%; 95% CI, 0%-1.4%, *P* = .06). Compared with patients in the nonexposed group, those in the exposed group had 2.2% fewer medication prescriptions filled per patient per quarter (absolute decrease, −0.2 prescriptions filled per patient per quarter; 95% CI, −0.4 to −0.1; *P* = .04; Table 2).

**Table 2.** Differential Changes in VA Facility Use Over Time Among Exposed vs Nonexposed Cohorts^**a**^

Time Since Storm^b^	Facility Use per Quarter					
	Primary Care Visit, % (95% CI)	*P* Value	Inpatient Admission, % (95% CI)	*P* Value	Medication Fills per Patient, No. (95% CI)	*P* Value
Baseline	47.8	NA	6.6	NA	9.1	NA
6 mo	−12.4 (−13.8 to −10.9)	<.001	−1.4 (−2.1 to −0.6)	<.001	−0.9 (−1.0 to −0.7)	<.001
12 mo	−1.6 (−2.8 to −0.4)	.01	0.5 (−0.3 to 1.2)	.21	−0.4 (−0.5 to −0.2)	<.001
18 mo	−0.1 (−1.5 to 1.4)	.94	0.7 (0.0 to 1.4)	.05	−0.2 (−0.4 to 0.1)	.04
24 mo	2.5 (1.1 to 3.9)	<.001	−0.3 (−1.1 to 0.4)	.42	−0.4 (−0.6 to −0.2)	<.001

Abbreviations: NA, not applicable; VA, Veterans Affairs.

^a^ Average differential changes in VA facility use outcomes were calculated using difference-in-differences regression analysis adjusted for individual fixed effects, between–zip code differences, and common time trends; 95% CIs were calculated using Huber-White robust SEs clustered at the zip code level.

^b^ Number of months after superstorm Sandy hit New York City on October 29, 2012.

### Health Outcomes

Before the storm, similar trends were observed in the control of BP among the exposed and nonexposed cohorts (Figure 1). The adjusted difference-in-differences estimate was not significantly different from 0 for all quarter-years before the storm, which supports the key identifying assumption of parallel trends (eTable 2 in the Supplement).

**Figure 2.**
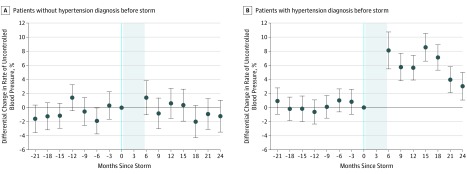
Differential Changes in Quarterly Rates of Uncontrolled Blood Pressure Among Exposed vs Nonexposed Cohorts by Subgroup With and Without a Hypertension Diagnosis Before Superstorm Sandy A, Patients without hypertension diagnosis before storm. B, Patients with hypertension diagnosis before storm. Points indicate the estimated average differential change between the exposed vs nonexposed groups in the percentage of patients with uncontrolled blood pressure in each quarter year since the storm, using a difference-in-differences regression analysis that adjusted for individual fixed effects, between–zip code differences, and common time trends. Vertical lines indicate the 95% CIs. The shaded area indicates the 6-month facility closure.

The prevalence of uncontrolled BP increased more in the exposed group than the nonexposed group in each quarter-year after the facility reopened (Table 3; Figure 2; eTable 2 in the Supplement). Compared with the nonexposed group, the adjusted change in uncontrolled BP among the exposed group, starting at a baseline mean (SD) of 19.3% (40.4%), was 6.5% greater at 6 months after the storm (95% CI, 4.5%-8.7%; unadjusted increase, 7.4% in the exposed group vs 2.1% in the nonexposed group), 4.5% greater at 12 months (95% CI, 3.1%-5.9%; *P* < .001; unadjusted increase, 5.3% vs 1.1%), 5.0% greater at 18 months (95% CI, 3.5%-6.0%; *P* < .001; unadjusted increase, 5.5% vs 1.3%), and 2.1% greater at 24 months (95% CI. 0.5%-3.6%; *P* < .001; unadjusted increase, 5.2% vs 3.5%). These values represent relative increases of 33.7%, 23.3%, 25.9%, and 10.7%, respectively, from the baseline rate of uncontrolled hypertension. This result was confined to individuals with a previous diagnosis of hypertension (Table 3; Figure 2; eTable 3 in the Supplement). No differential change was observed in BP control among individuals without a hypertension diagnosis before the storm (Figure 2; eTable 2 in the Supplement).

**Table 3.** Differential Changes in Health Outcomes Over Time Among Exposed vs Nonexposed Cohorts^**a**^

Time Since Storm^b^	Health Outcome, % (95% CI)											
	Uncontrolled Hypertension^c^	*P* Value	Systolic BP, mm Hg	*P* Value	Diastolic BP, mm Hg	*P* Value	Uncontrolled Diabetes^d^	*P* Value	Uncontrolled Cholesterol^e^	*P* Value	Patient Weight^f^	*P* Value
Baseline, mean (SD)	19.3 (40.4)	NA	127.8 (17.2)	NA	73.2 (11.6)	NA	15.2 (36.9)	NA	10.3 (30.3)	NA	193.8 (42.8)	NA
6 mo	6.5 (4.5 to 8.7)	<.001	3.8 (3.1 to 4.5)	<.001	2.7 (2.3 to 3.1)	<.001	1.9 (−0.1 to 4.0)	.11	1.3 (−0.1 to 2.6)	.11	−0.1 (−0.5 to 0.2)	.48
12 mo	4.5 (3.1 to 5.9)	<.001	2.3 (1.7 to 2.9)	<.001	2.2 (1.9 to 2.6)	<.001	1.7 (−0.3 to 3.6)	.15	0.6 (−0.6 to 1.8)	.40	0.2 (−0.2 to 0.5)	.38
18 mo	5.0 (3.5 to 6.5)	<.001	3.1 (2.5 to 3.7)	<.001	2.9 (2.5 to 3.3)	<.001	0.8 (−1.2 to 2.8)	.95	−0.7 (−2.0 to 0.6)	.37	−0.2 (−0.5 to 0.2)	.49
24 mo	2.1 (0.5 to 3.6)	.01	1.5 (0.9 to 2.1)	<.001	2.0 (1.7 to 2.4)	<.001	−0.2 (−2.2 to 1.8)	.87	−0.2 (−1.4 to 1.0)	.76	0.5 (0.1 to 0.9)	.02

Abbreviations: BP, blood pressure; NA, not applicable.

^a^ Average differential changes in health outcomes were calculated using difference-in-differences regression analysis adjusted for individual fixed effects, between–zip code differences, and common time trends; 95% CIs were calculated using Huber-White robust SEs clustered at the zip code level.

^b^ Number of months after superstorm Sandy hit New York City on October 29, 2012.

^c^ Uncontrolled hypertension was defined as a binary variable equal to 1 if a patient's mean BP was greater than 140/90 mm Hg in a given quarter-year and equal to 0 otherwise.

^d^ Uncontrolled diabetes was defined as a binary variable equal to 1 if a patient's mean hemoglobin A_1c_ was greater than 8% (to convert to proportion of hemoglobin, multiply by 0.01) in a given quarter-year and equal to 0 otherwise.

^e^ Uncontrolled cholesterol was defined as a binary variable equal to 1 if a patient's mean low-density lipoprotein was greater than 140 mg/dL [to convert to millimoles per liter, multiply by 0.0259] in a given quarter-year and equal to 0 otherwise.

^f^ Patient weight, expressed in pounds, was taken from electronic medical records. To convert pounds to kilograms, multiply the number of pounds by 0.45359237.

Among secondary health outcomes, both systolic and diastolic BP were higher in every quarter-year after the facility reopened. Three months after the facility reopened, rates of uncontrolled diabetes were 3.6% higher in the exposed cohort than the nonexposed cohort (95% CI, 1.9%-5.3%; *P* < .001; eTable 4 in the Supplement). At 6 months after reopening, the differential change in rates of uncontrolled diabetes was not statistically significant. No evidence of changes in cholesterol control or patient weight was observed (Table 3).

Among veterans jointly exposed to decreased health care access and flooding from the storm surge, a slightly larger change in the rate of uncontrolled BP was observed compared with veterans exposed only to decreased health care access; however, the difference was not statistically significant (eTable 3 in the Supplement).

The magnitude and statistical significance of the results were similar in each sensitivity analysis performed (eTable 5 and eTable 6 in the Supplement), including analyses that assumed the worst-case scenario for bias from differential attrition, that used a matched control group with characteristics more similar to the exposed group, and that adjusted for observable and unobservable time-varying geographic factors, such as exposure to the storm surge, which may have differed across groups.

## Discussion

Two years after a 6-month interruption in access to health care services owing to the temporary closure of the VA Manhattan Medical Center after superstorm Sandy, we observed persistently higher rates of poorly controlled hypertension among veterans exposed to the closure. These changes in chronic disease control followed a temporary period of sharply decreased rates of primary care visits and coincided with decreased medication prescription fills per quarter in the exposed cohort. Despite these changes, we did not observe clinically significant changes in diabetes control, lipid control, or patient weight. Our results suggest that temporary gaps in access to health care services may be associated with long-term negative changes in hypertension control but not in diabetes control, hyperlipidemia control, or patient weight.

Several possible mechanisms may explain the persistence of uncontrolled BP among exposed veterans after the facility reopened. Blood pressure control may have worsened among patients with diagnosed hypertension because they may have been less likely to fill their medication prescriptions in a timely manner when their usual site of care was unavailable. In support of this explanation, we observed a decreased rate of prescription fills per quarter among the exposed cohort; however, the magnitude of the decrease was likely too small to fully explain the results. In addition, with decreased access to primary care, these patients may also have delayed titration of their hypertension medications, leading to longer periods of uncontrolled BP. Another possibility is that the disruption of superstorm Sandy itself may have introduced financial and emotional stressors that changed patients’ control of their chronic illnesses. We find some support for this possibility in the observation that patients in flooded areas experienced a slightly larger increase in the rate of uncontrolled BP; however, the difference was not significant (eTable 3 in the Supplement), and no changes were observed to be associated with exposure to the storm surge alone (eTable 7 in the Supplement). Further, our results did not change after controlling for time-varying geographic factors that may have differed across groups, such as exposure to the storm surge.

There is no clear explanation for why these mechanisms would influence BP control but not diabetes or hyperlipidemia control. Changes in uncontrolled diabetes were observed 3 months after the facility reopened. Although point estimates remained positive over the subsequent 18 months, they were not statistically significant, partly owing to large standard errors. It is possible that we observed an attenuated effect in diabetes and cholesterol control because hemoglobin A_1c_ and low-density lipoprotein require relatively infrequent laboratory testing compared with BP and because fewer patients in the sample had diabetes and high cholesterol levels. Therefore, our study may have had insufficient statistical power to observe a small, persistent association between exposure to reduced access to care and diabetes and cholesterol control.

Our findings are consistent with short-term results from a study by Schaller and Stevens,^10^ which concluded that individuals with chronic conditions who were covered by employer-sponsored health insurance reported lower use of health care services and poorer self-reported health immediately after job loss. However, to our knowledge, long-term changes in chronic disease control after a temporary period of decreased access to health care have not previously been documented. Thus, we believe our findings address a recognized evidence gap related to the long-term health consequences of interruptions in health care access.^4^ To date, the literature has focused on either permanent shifts in access to care or immediate changes in health during gaps in health insurance coverage, limiting the ability to investigate long-term consequences of an episodic change.^1,2,4^ Because the decrease in health care access we studied was both temporary and known to be temporary at the time, it is unlikely that our results can be explained by confounding from anticipatory behavior.^27^

Beyond its examination of decreased health care access, our study contributes data on the association between long-term morbidity and natural disasters. The frequency of destructive hurricanes, such as superstorm Sandy, is expected to increase with climate change, and recent findings suggest there are large indirect mortality consequences of destructive hurricanes that are mediated in part by poor health care access.^28^ However, the associations between natural disasters and morbidity have not been sufficiently studied to date, even as the economic costs associated with morbidity are likely much larger than costs associated with mortality.^29,30,31^ Our findings suggest that fully capturing the association between natural disasters and morbidity rates will require accounting for patients’ exposure to disrupted health care access. Thus, the common practice of modeling exposure to natural disasters based solely on patients’ proximity to extreme meteorological conditions^32,33^ may systematically underestimate the health consequences of natural disasters.

### Limitations

This study had several important limitations. Owing to the temporary closure of the Manhattan VA facility, differential changes may have occurred in the exposed vs nonexposed cohorts after the storm. We found that the rate of follow-up and the demographic characteristics of patients who received follow-up did not differ across cohorts over time. Further, we used individual fixed effects to control for unobservable individual factors that may have been associated with attrition. Nonetheless, our results may have been biased by unobserved compositional changes in patient characteristics associated with exposure. We also performed several sensitivity analyses, including analyses using alternate control groups with characteristics more similar to the exposed group, restricting analyses to balanced panels of patients with 1 or more VA appointments, BP measurement, hemoglobin A_1c_ value, lipid panel, and recorded patient weight, respectively, both before and after the storm, and we estimated results by assuming the worst-case scenario for bias from attrition, which did not meaningfully change the findings. In addition, we only observed visits to VA facilities. Patients may have substituted non-VA health care services for VA health care services during the 6-month closure, however this would have likely biased our findings against rejecting the null hypothesis. Finally, our analysis focused on a population of veterans who were predominantly male and older in comparison with the overall adult population with hypertension in the United States.^34^

## Conclusions

We observed an association between a temporary decrease in health care access and a long-term increase in the rate of uncontrolled hypertension but not in the rates of uncontrolled diabetes, uncontrolled hyperlipidemia, or increased patient weight. Our analysis may have lacked the statistical power needed to identify smaller changes in the control of chronic illnesses, such as diabetes and hyperlipidemia, for which outcomes are less frequently assessed than for hypertension. Our results suggest that reducing temporary gaps in health care access, either by preventing disruptions when possible or by providing increased services to those exposed to decreased access to care, may be an important target for population health improvements.

## Notes

Supplement. **eMethods.** Main Specification and Methods to Assess Effects of Joint Exposure to Reduced Access and Storm Surge **eFigure 1.** Exposure to Reduced Access to Health Care Versus Flooding, By Zip Code **eFigure 2.** Changes in Sample Composition Associated With Reduced Access to Care **eFigure 3.** Monthly Visit Count at the VA Manhattan Medical Center **eTable 1.** Changes in Utilization Associated With Reduced Access to Care **eTable 2.** Changes in Uncontrolled Blood Pressure Associated With Reduced Access to Care **eTable 3.** Heterogeneous Changes in Blood Pressure Control Associated With Reduced Access to Care **eTable 4.** Changes in Secondary Health Outcomes Associated With Reduced Access to Care **eTable 5.** Sensitivity Analyses for the Primary Outcome **eTable 6.** Sensitivity Analyses for Secondary Outcomes **eTable 7.** Changes in Outcomes Associated With Flooding From the Storm
