# Supplementary material for: Association Between a Temporary Reduction in Access to Health Care and Long-term Changes in Hypertension Control Among Veterans After a Natural Disaster
Source: JAMA Netw Open. 2019 Nov 13;2(11):e1915111. doi: 10.1001/jamanetworkopen.2019.15111 (PMC6902789; doi:10.1001/jamanetworkopen.2019.15111)
Supplement: Supplement. — eMethods. Main Specification and Methods to Assess Effects of Joint Exposure to Reduced Access and Storm Surge eFigure 1. Exposure to Reduced Access to Health Care Versus Flooding, By Zip Code eFigure 2. Changes in Sample Composition Associated With Reduced Access to Care eFigure 3. Monthly Visit Count at the VA Manhattan Medical Center eTable 1. Changes in Utilization Associated With Reduced Access to Care eTable 2. Changes in Uncontrolled Blood Pressure Associated With Reduced Access to Care eTable 3. Heterogeneous Changes in Blood Pressure Control Associated With Reduced Access to Care eTable 4. Changes in Secondary Health Outcomes Associated With Reduced Access to Care eTable 5. Sensitivity Analyses for the Primary Outcome eTable 6. Sensitivity Analyses for Secondary Outcomes eTable 7. Changes in Outcomes Associated With Flooding From the Storm [file jamanetwopen-2-e1915111-s001.pdf]

Supplementary Online Content

Baum A, Barnett ML, Wisnivesky J, Schwartz MD. Association between a temporary reduction in access to health care and long-term changes in hypertension control among veterans after a natural disaster. *JAMA Netw Open*. 2019;2(11):e1915111. doi:10.1001/jamanetworkopen.2019.15111

eMethods. Main Specification and Methods to Assess Effects of Joint Exposure to Reduced Access and Storm Surge

eFigure 1. Exposure to Reduced Access to Health Care Versus Flooding, By Zip Code

eFigure 2. Changes in Sample Composition Associated With Reduced Access to Care

eFigure 3. Monthly Visit Count at the VA Manhattan Medical Center

eTable 1. Changes in Utilization Associated With Reduced Access to Care

eTable 2. Changes in Uncontrolled Blood Pressure Associated With Reduced Access to Care

eTable 3. Heterogeneous Changes in Blood Pressure Control Associated With Reduced Access to Care

eTable 4. Changes in Secondary Health Outcomes Associated With Reduced Access to Care

eTable 5. Sensitivity Analyses for the Primary Outcome

eTable 6. Sensitivity Analyses for Secondary Outcomes

eTable 7. Changes in Outcomes Associated With Flooding From the Storm

This supplementary material has been provided by the authors to give readers additional information about their work.

© 2019 Baum A et al. *JAMA Network Open*.

eMethods. Main Specification and Methods to Assess Effects of Joint Exposure to Reduced Access and Storm Surge

Main Specification

The specification for our main analysis was

$$y_{i,t} = \alpha_t Closure_{i,t} + \theta_i + \theta_z + \theta_t + \varepsilon_i$$

where $y_{i,t}$ is health outcome of individual *i* in quarter-year *t* (e.g. uncontrolled blood pressure is a binary variable equal to 1 if patient *i's* mean blood pressure exceeds 140/90 mmHg in a given quarter-year, and 0 otherwise); $Closure_{i,t} = 1$ when *t* > October 29, 2012 and patient *i* was attributed to the VA Manhattan cohort prior to the storm, and 0 otherwise; and θ_i, θ_z and θ_t are individual, zip code, and quarter-year effects.

Methods to Assess Effects of Joint Exposure to Reduced Access and Storm Surge

Defining Exposed and Control Groups

In secondary analyses, we examined effects on the subpopulation exposed to reduced access as well as flooding from the storm surge caused by Sandy. The storm surge destroyed thousands of homes and left millions of people across multiple states without electricity,[23] causing quasi-random variation in household flooding. There were 10,826 individuals attributed to the Manhattan VA prior to the storm who lived in a zip code that was exposed to flooding. To determine exposure to the storm surge, a zip code level indicator was calculated based on the Federal Emergency Management Agency Modeling Task Force's determination of the extent of water inundation.[35] We overlaid this exposure map with zip code boundaries to generate a dummy variable = 1 if a patient's zip code of residence had any spatial exposure to the surge versus no exposure (**eFigure 1**). The control population – those not exposed to reduced access nor to flooding – consisted of 28,783 individuals attributed to either the Bronx, NY, Brooklyn, NY or West Haven, CT VA who lived in a zip code that was not exposed to flooding.

Statistical Analysis

We used the same difference-in-differences analysis[24] as in the main analysis to compare quarterly within-subject changes in each dependent variable over the two years before and after the storm among patients exposed to both reduced access to care and the storm surge, relative to changes over the same time period among patients exposed to neither. The key independent

© 2019 Baum A et al. *JAMA Network Open*.

variables in the model are interactions between joint exposure (Manhattan VA attribution and storm surge exposure) and each quarter-year time period. The estimated coefficients on these interaction terms describes the average differential change in the outcome for jointly-exposed patients vs. non-exposed patients over time. We included individual fixed effects, quarter-year fixed effects and zip code of residence fixed effects. Huber-White robust standard errors were clustered at the zip code level.

© 2019 Baum A et al. *JAMA Network Open*.

eFigure 1. Exposure to Reduced Access to Health Care Versus Flooding, By Zip Code

Panel A. Exposure to Reduced Access to Care

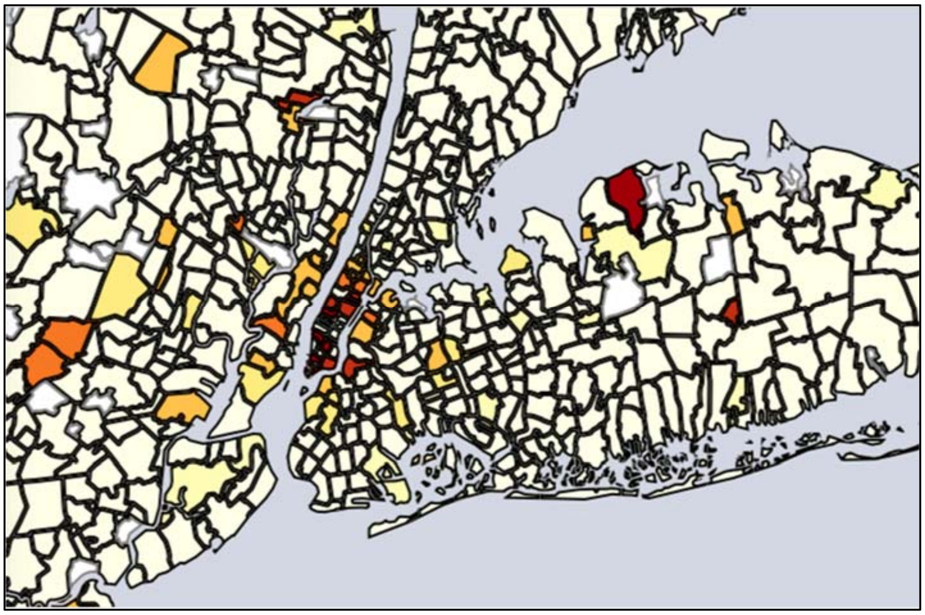

Panel B. Exposure to Storm Flooding

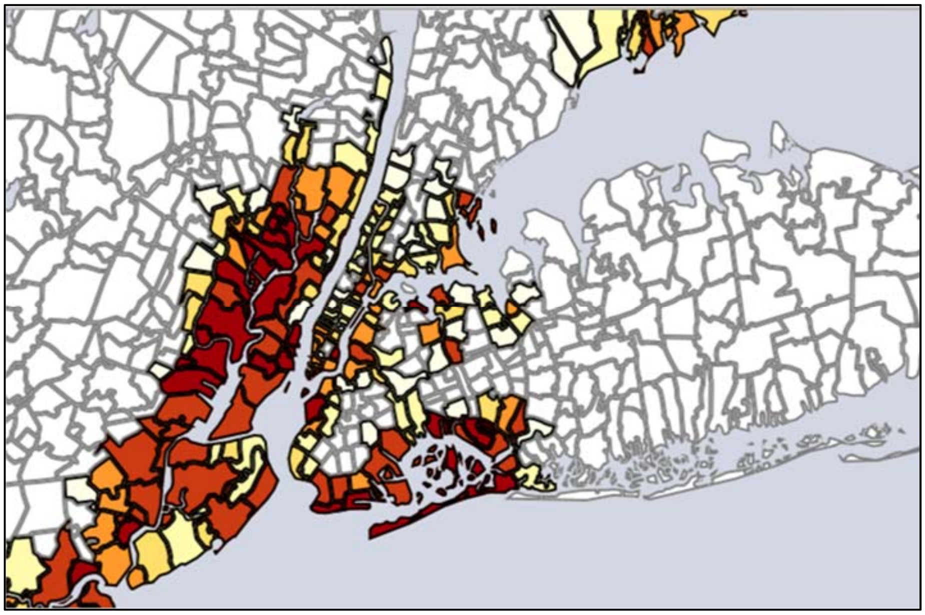

A. Exposure to Reduced Access to Care: Darker colors indicate a greater number of individuals in the zip code were attributed to the VA Manhattan Medical Center prior to the storm.

B. Exposure to Zip Code Flooding: Darker colors indicate greater extent of flooding. Zip code flooding level was estimated based on the Federal Emergency Management Agency Modeling Task Force's determination of the extent of inundation. Their determination was based on U.S. Geological Survey

© 2019 Baum A et al. *JAMA Network Open*.

field-verified High-Water Marks and Storm Surge Sensor data, which were used to interpolate a water surface elevation, then subtracted from the best available Digital Elevation Model to create a depth grid and surge boundary. We overlaid this exposure map with zip code boundaries to generate a dummy variable = 1 if a patient's zip code of residence had any spatial exposure to the surge.

© 2019 Baum A et al. *JAMA Network Open*.

eFigure 2. Changes in Sample Composition Associated With Reduced Access to Care

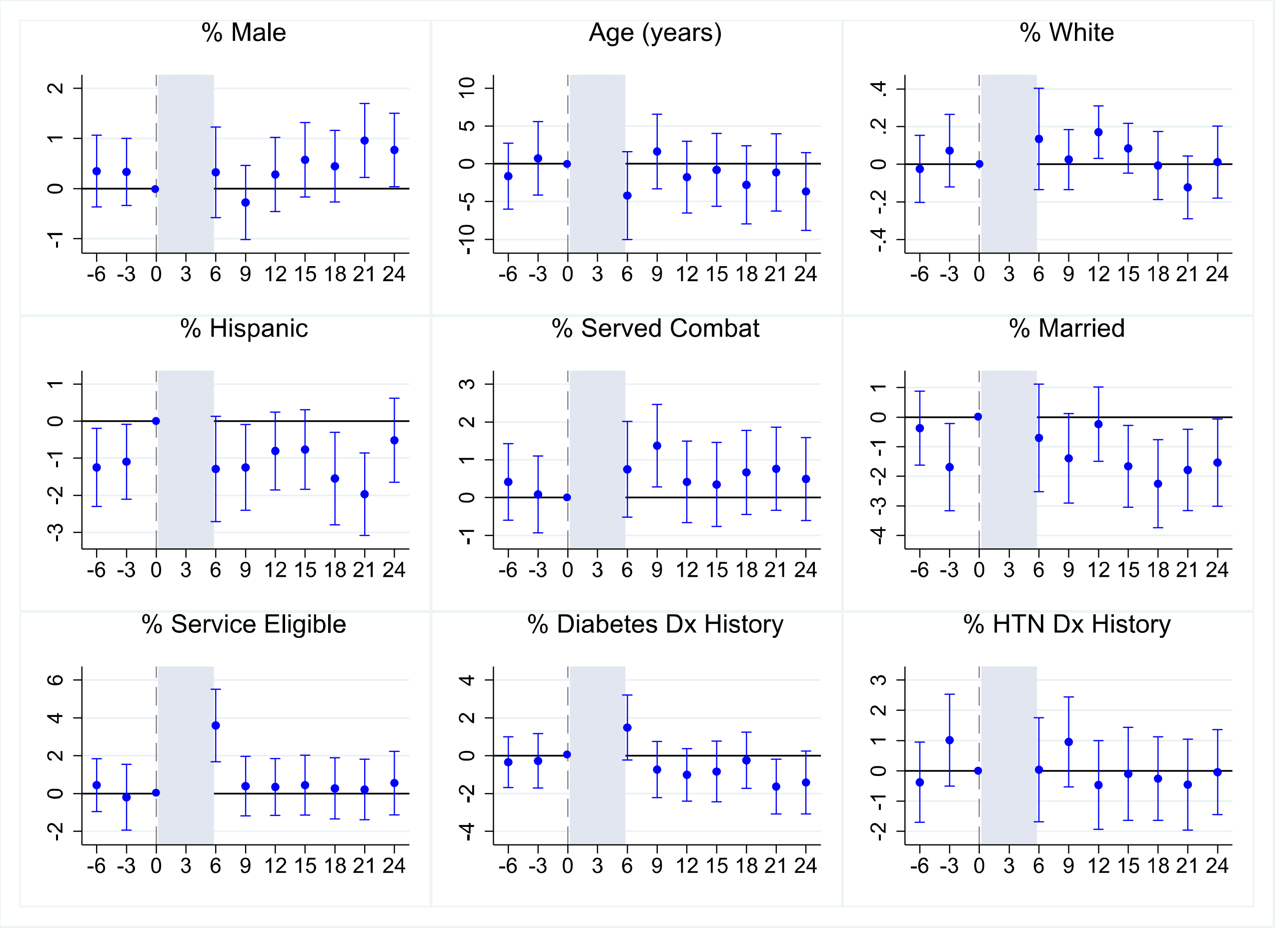

Average change by quarter-year in the pre-storm demographic characteristics and diagnosis histories of the sample from the main difference-in-differences regression analysis with adjustment between-zip code differences and common time trends. 95% confidence intervals were calculated using Huber-White robust standard errors clustered at the zip code level.

© 2019 Baum A et al. *JAMA Network Open*.

eFigure 3. Monthly Visit Count at the VA Manhattan Medical Center

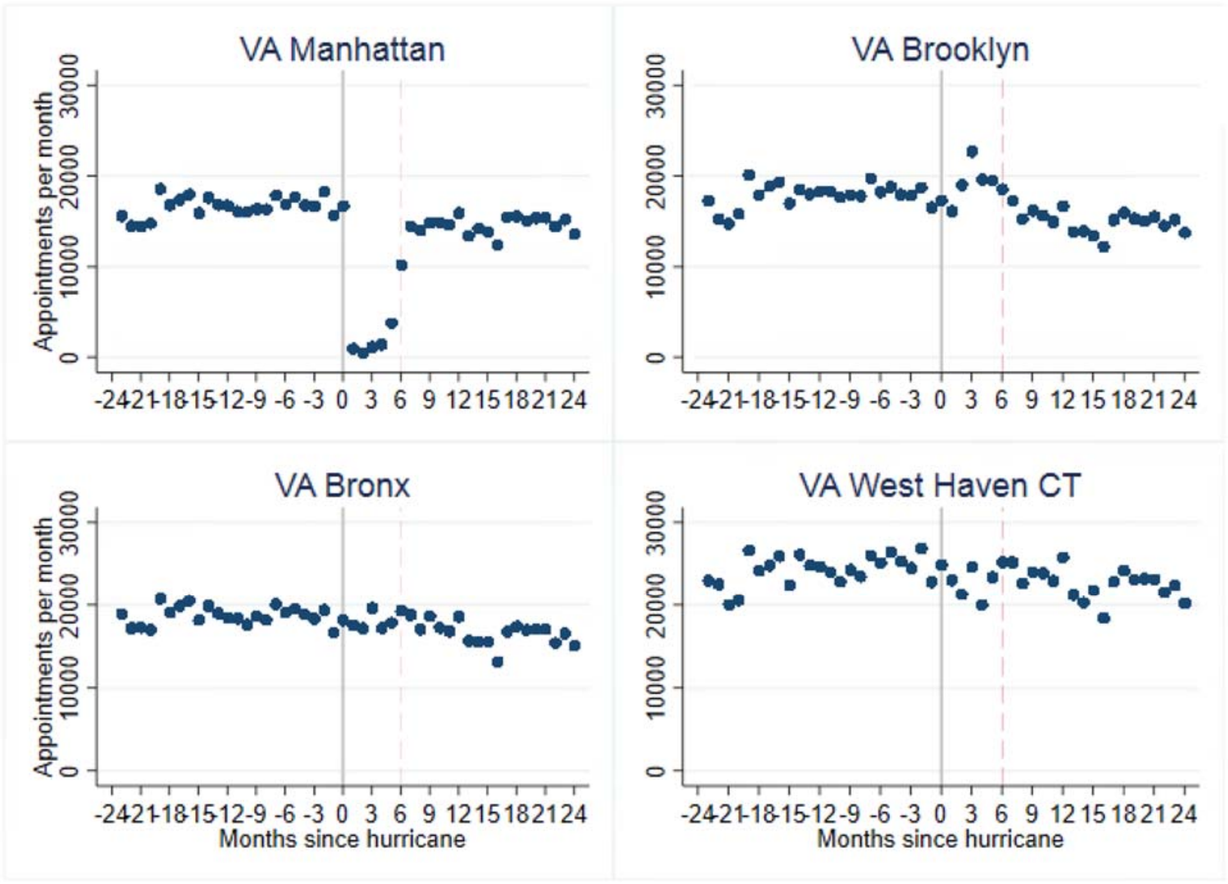

© 2019 Baum A et al. *JAMA Network Open*.

eTable 1. Changes in Utilization Associated With Reduced Access to Care

	**Primary Care Visits** (% patients with 1 or more)	**IP Admissions** (% patients with 1 or more)	**Medication fills per patient** (no.)
-4Q X Exposure	0.04	0.14	-0.06
	(0.63)	(0.33)	(0.09)
-3Q X Exposure	0.49	0.15	-0.16
	(0.78)	(0.36)	(0.09)
-2Q X Exposure	-0.62	0.21	-0.21*
	(0.66)	(0.33)	(0.09)
-1Q X Exposure	2.48***	0.61	-0.18*
	(0.68)	(0.33)	(0.08)
+1Q X Exposure	-24.77***	-2.42***	-0.68***
	(0.88)	(0.31)	(0.09)
+2Q X Exposure	-12.36***	-1.35***	-0.85***
	(0.73)	(0.37)	(0.08)
+3Q X Exposure	0.36	-0.33	-0.25**
	(0.71)	(0.32)	(0.10)
+4Q X Exposure	-1.60*	0.46	-0.35***
	(0.62)	(0.37)	(0.10)
+5Q X Exposure	2.01**	0.87*	-0.15
	(0.64)	(0.38)	(0.10)
+6Q X Exposure	-0.06	0.69	-0.19*
	(0.76)	(0.36)	(0.09)
+7Q X Exposure	2.67***	0.39	-0.18
	(0.68)	(0.36)	(0.10)
+8Q X Exposure	2.49***	-0.30	-0.37***
	(0.70)	(0.38)	(0.10)
Patient FE	Yes	Yes	Yes
Quarter-Year FE	Yes	Yes	Yes
Zip code FE	Yes	Yes	Yes
Observations	764,447	786,621	598,307
R-squared	0.26	0.28	0.72

Cluster-robust standard errors in parentheses.
* *P*<0.05, ** *P*<0.01, *** *P*<0.001

Average change by quarter-year in the percent of patients with at least one visit to a primary care provider per quarter, the percent of patients with at least one inpatient admission per quarter, and the count of prescription medication fills from the main difference-in-differences regression analysis with adjustment for individual fixed effects, between-zip code differences, and common time trends. 95% confidence intervals were calculated using Huber-White robust standard errors clustered at the zip code level. FE is "Fixed Effects".

© 2019 Baum A et al. *JAMA Network Open*.

eTable 2. Changes in Uncontrolled Blood Pressure Associated With Reduced Access to Care

	**Overall** (% Change in Poor BP Control)	**Baseline HTN** (% Change in Poor BP Control)	**No Baseline HTN** (% Change in Poor BP Control)
-4Q X Exposure	-0.22	-0.63	1.39
	(0.69)	(0.87)	(0.95)
-3Q X Exposure	-0.12	0.10	-0.56
	(0.68)	(0.82)	(0.99)
-2Q X Exposure	0.31	1.01	-1.90*
	(0.68)	(0.83)	(0.94)
-1Q X Exposure	0.65	0.82	0.29
	(0.74)	(0.90)	(0.99)
+1Q X Exposure	15.27***	18.46***	4.12
	(1.70)	(2.01)	(2.67)
+2Q X Exposure	6.54***	8.11***	1.40
	(1.03)	(1.34)	(1.24)
+3Q X Exposure	4.29***	5.75***	-0.83
	(0.78)	(0.99)	(1.11)
+4Q X Exposure	4.50***	5.66***	0.59
	(0.73)	(0.89)	(1.08)
+5Q X Exposure	6.66***	8.55***	0.35
	(0.83)	(1.00)	(1.16)
+6Q X Exposure	5.01***	7.11***	-1.99
	(0.77)	(0.91)	(1.17)
+7Q X Exposure	2.83***	3.95***	-0.92
	(0.77)	(0.94)	(1.11)
+8Q X Exposure	2.05**	3.03**	-1.22
	(0.81)	(1.00)	(1.15)
Patient FE	Yes	Yes	Yes
Quarter-Year FE	Yes	Yes	Yes
Zip code FE	Yes	Yes	Yes
Observations	353,511	274,428	79,083
R-squared	0.38	0.36	0.37

Cluster-robust standard errors in parentheses.
* P<0.05, ** P<0.01, *** P<0.001

Average change by quarter-year in systolic blood pressure from the main difference-in-differences regression analysis with adjustment for individual fixed effects, between-zip code differences, and common time trends. 95% confidence intervals were calculated using Huber-White robust standard errors clustered at the zip code level. FE is "Fixed Effects".

© 2019 Baum A et al. *JAMA Network Open*.

eTable 3. Heterogeneous Changes in Blood Pressure Control Associated With Reduced Access to Care

	Subgroup Indicator		
	Pre-storm Diagnosis of Hypertension	Age > 65 years	Exposed to Storm Surge
Exposure X Post	0.28	3.15***	3.97***
	(0.45)	(0.44)	(0.59)
Exposure X Post X Subgroup Indicator	5.73***	3.23***	1.06
	(0.58)	(0.68)	(0.72)
Quarter-Year FE	Yes	Yes	Yes
Zip code FE	Yes	Yes	Yes
Patient FE	Yes	Yes	Yes
Observations	353,511	353,511	351,057

Cluster-robust standard errors in parentheses.
[*] $P<0.05$, [**] $P<0.01$, [***] $P<0.001$

Average change blood pressure control associated with exposure from a pre-post difference-in-differences regression analysis with adjustment for individual fixed effects, between-zip code differences, and common time trends. 95% confidence intervals were calculated using Huber-White robust standard errors clustered at the zip code level. FE is "Fixed Effects".

© 2019 Baum A et al. *JAMA Network Open*.

eTable 4. Changes in Secondary Health Outcomes Associated With Reduced Access to Care

	**Systolic BP** (mmHg)	**Diastolic BP** (mmHg)	**Uncontrolled Diabetes** (%)	**Uncontrolled Cholesterol** (%)	**Weight** (lbs)
-4Q X Exposure	-0.98**	-1.24***	2.43*	-0.72	0.87
	(0.25)	(0.17)	(0.99)	(0.56)	(0.17)
-3Q X Exposure	0.19	-0.21	1.79	-1.48	0.31
	(0.26)	(0.18)	(0.93)	(0.57)	(0.16)
-2Q X Exposure	0.34	0.15	1.31	-0.11	-0.06
	(0.26)	(0.16)	(0.97)	(0.58)	(0.14)
-1Q X Exposure	0.05	-0.14	1.63	0.52	-0.10
	(0.28)	(0.16)	(0.96)	(0.61)	(0.11)
+1Q X Exposure	6.91***	4.23***	3.52**	1.10	0.04
	(0.63)	(0.38)	(1.23)	(0.90)	(0.19)
+2Q X Exposure	3.78***	2.68***	1.94	1.26	-0.14
	(0.34)	(0.22)	(1.05)	(0.69)	(0.18)
+3Q X Exposure	2.38 ***	1.05***	3.62***	1.14	0.12
	(0.28)	(0.17)	(0.88)	(0.60)	(0.16)
+4Q X Exposure	2.28***	2.24***	1.66	0.57	0.15
	(0.29)	(0.17)	(0.98)	(0.60)	(0.16)
+5Q X Exposure	3.85***	3.29***	0.80	-1.71*	-0.12
	(0.30)	(0.20)	(0.98)	(0.68)	(0.18)
+6Q X Exposure	3.12***	2.90***	0.78	-0.7	-0.15
	(0.30)	(0.19)	(1.02)	(0.68)	(0.20)
+7Q X Exposure	2.16***	2.24***	1.10	-1.12	0.34
	(0.29)	(0.17)	(1.07)	(0.64)	(0.20)
+8Q X Exposure	1.51***	2.02***	-0.20	-0.20	0.48*
	(0.31)	(0.19)	(1.02)	(0.61)	(0.19)
Patient FE	Yes	Yes	Yes	Yes	Yes
Quarter-Year FE	Yes	Yes	Yes	Yes	Yes
Zip code FE	Yes	Yes	Yes	Yes	Yes
Observations	353,567	353,567	158,225	248,543	482,500
R-squared	0.53	0.60	0.61	0.53	0.96

Cluster-robust standard errors in parentheses.
* P<0.05, ** P<0.01, *** P<0.001

Average change by quarter-year in systolic and diastolic blood pressure (mmHg), poor diabetes control (% patients with HbA1C > 8%), poor cholesterol control (% patients with low-density lipoprotein > 140 mg/dl), and weight (lbs.), from the main difference-in-differences regression analysis with adjustment for individual fixed effects, between-zip code differences, and common time trends. 95% confidence intervals were calculated using Huber-White robust standard errors clustered at the zip code level. FE is "Fixed Effects".

© 2019 Baum A et al. *JAMA Network Open*.

eTable 5. Sensitivity Analyses for the Primary Outcome

Panel A. Alterations to the Sample

	Main	Lee Bounds	Balanced visits sample	Balanced BP sample	Matched Controls 1:1 CEM	Drop West Haven VA	Attribution based on >50% pre-storm visits	Attribution based on plurality of pre-storm visits
Treated X Post	4.74***	2.56***	4.74***	4.72***	4.86***	4.28***	4.97***	4.06***
	(0.35)	(0.34)	(0.35)	(0.35)	(0.47)	(0.38)	(0.38)	(0.57)
Quarter-Year FE	Yes	Yes	Yes	Yes	Yes	Yes	Yes	Yes
Zip code FE	Yes	Yes	Yes	Yes	Yes	Yes	Yes	Yes
Patient FE	Yes	Yes	Yes	Yes	Yes	Yes	Yes	Yes
Observations	353,511	346,604	342,023	323,912	128,433	222,988	280,542	285,970

Cluster-robust standard errors in parentheses.
* P<0.05, ** P<0.01, *** P<0.001

Lee bounds is a procedure that assumes the excess attrition within the exposed cohort is caused by the departure of the healthiest patients from the sample (mechanically, it trims the control group at the quantile of the outcome variable that corresponds to the share of excess observations in the control group relative to the treatment group). *Balanced visit sample* restricts analysis to the subgroup of patients who had a visit to any VHA facility both before and after the hurricane. *Balanced BP sample* restricts analysis to the subgroup of patients who had a blood pressure recorded at a VHA facility both before and after the hurricane. *Matched Controls* constructed a matched control group by sampling from patients within the overall control group with 1:1 coarsened exact matching by age, sex, race, married status, combat history, VA eligibility, zip code exposure to storm surge, and pre-storm diagnoses history of hypertension and diabetes to generate a control group similar to the treated group. *Drop West Haven VA* uses only patients attributed to the Manhattan VA, Bronx VA, or Brooklyn VA (excluding patients attributed to the West Haven, CT VA. *FE* is "Fixed Effects".

© 2019 Baum A et al. *JAMA Network Open*.

Panel B. Alterations to Variables or Statistical Model

	Main	Two BP Measures > 140/90 mmHg	Drop Patient FE	Add Zip code × Quarter-year FE	Logistic Regression	Mixed Effects Model	Wild Bootstrap-t
Treated X Post	4.74***	5.47***	4.26***	3.72***	1.63*** (odds-ratio)	4.04***	4.74**
	(0.35)	(0.59)	(0.35)	(0.50)	(0.04)	(0.30)	*10,000 replications*
Quarter-Year FE	Yes	Yes	Yes	Yes	Yes	Yes	Yes
Zip code FE	Yes	Yes	Yes	Yes	Yes	No	Yes
Patient FE	Yes	Yes	No	Yes	Yes	No	Yes
Zip code × Quarter-Year FE	No	No	No	Yes	No	No	No
Observations	353,511	219,377	361,888	353,511	353,511	362,587	353,511

Cluster-robust standard errors in parentheses.
* *P*<0.05, ** *P*<0.01, *** *P*<0.001

Two BP Measures defines the binary primary dependent outcome variable equal to 1 (for uncontrolled blood pressure) if the patient had at least two BP measures > 140/90 mmHg recorded within a six-month period. *Zip code × Time FE* flexibly controls for time-varying geographic factors that may have differed across the exposed and control group, such as exposure to the storm surge. *Wild bootstrap-t* procedure with Webb Weights provides more conservative standard errors. *Mixed Effects Model* is a multilevel mixed-effects generalized linear model with patient-level random intercepts and fixed effects for quarter-year. *FE* is "Fixed Effects".

© 2019 Baum A et al. *JAMA Network Open*.

eTable 6. Sensitivity Analyses for Secondary Outcomes

Panel A. Balanced visit sample

Outcome Measure	6 months	12 months	18 months	24 months
**Uncontrolled Diabetes (%)**	1.8	1.5	-0.1	-0.3
**(95% CI)**	(-0.3, 3.8)	(-0.4, 3.5)	(-2.1, 2.0)	(-2.3, 1.6)
**P Value**	0.086	0.122	0.957	0.743
**Uncontrolled Cholesterol (%)**	1.3	0.6	-0.6	-0.1
**(95% CI)**	(-0.1, 2.7)	(-0.5, 1.8)	(-1.9, 0.7)	(-1.4, 1.1)
**P Value**	0.061	0.291	0.347	0.816
**Weight (lbs.)**	-0.1	0.2	-0.1	0.5
**(95% CI)**	(-0.5, 0.3)	(-0.1, 0.5)	(-0.5, 0.3)	(0.1, 0.9)
**P Value**	0.571	0.261	0.542	0.007

Panel B. Balanced outcome sample

Outcome Measure	6 months	12 months	18 months	24 months
**Uncontrolled Diabetes (%)**	2.2	1.9	0.1	-0.2
**(95% CI)**	(0.0, 4.4)	(-0.2, 3.9)	(-2.0, 2.3)	(-2.4, 1.8)
**P Value**	0.047	0.075	0.897	0.785
**Uncontrolled Cholesterol (%)**	1.3	0.5	-0.9	-0.2
**(95% CI)**	(-0.1, 2.6)	(-0.7, 1.7)	(-2.2, 0.4)	(-1.4, 1.0)
**P Value**	0.067	0.428	0.171	0.764
**Weight (lbs.)**	-0.1	0.2	-0.1	0.6
**(95% CI)**	(-0.5, 0.2)	(-0.1, 0.5)	(-0.5, 0.3)	(0.2, 0.9)
**P Value**	0.569	0.251	0.613	0.004

Cluster-robust standard errors in parentheses.
* P<0.05, ** P<0.01, *** P<0.001

Caption: Tables report the coefficient on Treated X Post for each outcome over time. *Panel A. Balanced visit sample* restricts analysis to the subgroup of patients who had any visit at a VHA facility both before and after the hurricane. *Panel B. Balanced outcome sample* restricts analysis to the subgroup of patients who had a value of the outcome recorded at a VHA facility both before and after the hurricane. *FE* is "Fixed Effects".

© 2019 Baum A et al. *JAMA Network Open*.

eTable 7. Changes in Outcomes Associated With Flooding From the Storm

Panel A. Utilization Outcomes

	Exposure to Storm Flooding Alone			*Joint Exposure to Access Reduction and Storm Flooding*		
	**Primary Care Visits** (% patients with 1 or more)	**IP Admissions** (% patients with 1 or more)	**Medication fills per patient** (no.)	**Primary Care Visits** (% patients with 1 or more)	**IP Admissions** (% patients with 1 or more)	**Medication fills per patient** (no.)
-4Q X Exposure	-1.04	0.59*	-0.03	-0.75	0.41	-0.11
	(0.57)	(0.28)	(0.08)	(1.00)	(0.42)	(0.10)
-3Q X Exposure	-1.03	0.48	0.12	0.537	0.19	-0.11
	(0.67)	(0.27)	(0.07)	(0.88)	(0.48)	(0.09)
-2Q X Exposure	-1.74	0.31	0.08	-0.80	0.19	-0.10
	(0.63)	(0.30)	(0.06)	(0.92)	(0.42)	(0.11)
-1Q X Exposure	-0.43	0.20	-0.12	3.19**	1.10**	-0.14
	(0.69)	(0.28)	(0.08)	(0.92)	(0.41)	(0.09)
+1Q X Exposure	-2.96*	0.02	-0.08	-26.45***	-2.29***	-0.79***
	(1.30)	(0.31)	(0.08)	(1.12)	(0.41)	(0.08)
+2Q X Exposure	-1.63*	0.28	-0.12	-13.43***	-0.98*	-0.99***
	(0.72)	(0.30)	(0.07)	(1.00)	(0.49)	(0.08)
+3Q X Exposure	-0.29	0.06	0.04	1.18	-0.22	-0.23*
	(0.65)	(0.30)	(0.07)	(0.88)	(0.40)	(0.10)
+4Q X Exposure	0.21	0.07	-0.03	-1.13*	0.68	-0.37***
	(0.62)	(0.31)	(0.09)	(0.83)	(0.50)	(0.09)
+5Q X Exposure	0.49	-0.78*	0.05	3.63***	1.26**	-0.12
	(0.62)	(0.28)	(0.09)	(0.93)	(0.46)	(0.11)
+6Q X Exposure	0.31	-0.61	0.12	0.87	1.44**	-0.03
	(0.69)	(0.29)	(0.09)	(1.02)	(0.44)	(0.11)
+7Q X Exposure	1.20	-0.37	0.04	4.65***	1.18**	-0.09
	(0.67)	(0.28)	(0.09)	(0.91)	(0.45)	(0.11)
+8Q X Exposure	0.79	-0.34	0.11	3.38***	0.96*	-0.39***
	(0.69)	(0.28)	(0.08)	(0.94)	(0.46)	(0.11)
Patient FE	Yes	Yes	Yes	Yes	Yes	Yes
Quarter-Year FE	Yes	Yes	Yes	Yes	Yes	Yes
Zip code FE	Yes	Yes	Yes	Yes	Yes	Yes
Observations	758,751	780,483	594,441	353,624	360,00	266,868
R-squared	0.24	0.28	0.72	0.26	0.42	0.72

Cluster-robust standard errors in parentheses.
[*] P<0.05, [**] P<0.01, [***] P<0.001

Results of difference-in-differences analysis comparing quarterly within-subject changes in each dependent variable over the two years before and after the storm among patients exposed to the storm surge alone, relative to changes over the same time period among patients not exposed to the storm surge. The key independent variables in the model are interactions between storm surge exposure and each quarter-year time period, with adjustment for individual fixed effects, between-zip code differences, and common time trends. 95% confidence intervals were calculated using Huber-White robust standard errors clustered at the zip code level. FE = "Fixed Effects".

© 2019 Baum A et al. *JAMA Network Open*.

Panel B. Changes in Uncontrolled Blood Pressure

	Exposure to Storm Alone			*Joint Exposure to Access Shock and Storm*		
	**Overall** (% Change in Poor BP Control)	**Baseline HTN** (% Change in Poor BP Control)	**No Baseline HTN** (% Change in Poor BP Control)	**Overall** (% Change in Poor BP Control)	**Baseline HTN** (% Change in Poor BP Control)	**No Baseline HTN** (% Change in Poor BP Control)
-4Q X Exposure	-0.48	-0.24	0.21	-0.50	-1.01	1.55
	(0.70)	(0.75)	(0.94)	(1.05)	(1.14)	(1.31)
-3Q X Exposure	0.06	-0.97	-1.13	-0.37	-0.64	0.82
	(0.67)	(0.83)	(1.07)	(0.90)	(1.09)	(1.35)
-2Q X Exposure	-0.13	0.37	-1.31	0.70	1.64	-2.64*
	(0.66)	(0.80)	(1.08)	(0.92)	(1.10)	(1.33)
-1Q X Exposure	0.86	-0.05	-0.33	0.41	0.61	0.23
	(0.82)	(0.80)	(0.93)	(1.00)	(1.24)	(1.22)
+1Q X Exposure	-0.06	1.15	0.17	17.70***	20.87***	6.48
	(0.82)	(0.95)	(1.26)	(2.37)	(2.72)	(3.80)
+2Q X Exposure	0.23	0.23	-1.36	6.62***	8.32***	0.75
	(0.76)	(0.95)	(1.03)	(1.39)	(1.74)	(1.48)
+3Q X Exposure	0.22	-0.07	1.51	4.30***	5.23***	1.17
	(0.68)	(0.84)	(1.08)	(1.02)	(1.31)	(1.36)
+4Q X Exposure	0.16	0.27	0.20	4.78***	5.86***	1.17
	(0.72)	(0.92)	(1.06)	(1.02)	(1.28)	(1.46)
+5Q X Exposure	0.17	0.46	1.06	6.58***	8.66***	-0.69
	(0.78)	(0.92)	(1.17)	(1.15)	(1.42)	(1.59)
+6Q X Exposure	0.78	1.24	-0.94	5.77***	8.13***	-2.44
	(0.73)	(0.90)	(1.14)	(1.04)	(1.27)	(1.59)
+7Q X Exposure	0.86	1.23	-0.52	3.38***	4.63***	-0.89
	(0.69)	(0.84)	(1.10)	(1.05)	(1.28)	(1.51)
+8Q X Exposure	0.64	0.54	0.86	2.81**	3.61**	-0.03
	(0.78)	(0.94)	(1.15)	(1.06)	(1.38)	(1.54)
Patient FE	Yes	Yes	Yes	Yes	Yes	Yes
Quarter-Year FE	Yes	Yes	Yes	Yes	Yes	Yes
Zip code FE	Yes	Yes	Yes	Yes	Yes	Yes
Observations	351,057	272,625	78,432	161,270	123,517	37,753
R-squared	0.38	0.36	0.37	0.38	0.36	0.38

Cluster-robust standard errors in parentheses.
[*] P<0.05, [**] P<0.01, [***] P<0.001

Results of difference-in-differences analysis comparing quarterly within-subject changes in each dependent variable over the two years before and after the storm among patients exposed to both reduced access to care and the storm surge, relative to changes over the same time period among patients exposed to neither. The key independent variables in the model are interactions between joint exposure (Manhattan VA attribution and storm surge exposure) and each quarter-year time period, with adjustment

© 2019 Baum A et al. *JAMA Network Open*.

for individual fixed effects, between-zip code differences, and common time trends. 95% confidence intervals were calculated using Huber-White robust standard errors clustered at the zip code level. FE = "Fixed Effects".

© 2019 Baum A et al. *JAMA Network Open*.
